# Spatial Transcriptome Profiling of Mouse Hippocampal Single Cell Microzone in Parkinson’s Disease

**DOI:** 10.3390/ijms24031810

**Published:** 2023-01-17

**Authors:** Erteng Jia, Yuqi Sheng, Huajuan Shi, Ying Wang, Ying Zhou, Zhiyu Liu, Ting Qi, Min Pan, Yunfei Bai, Xiangwei Zhao, Qinyu Ge

**Affiliations:** 1 State Key Laboratory of Bioelectronics, School of Biological Science & Medical Engineering, Southeast University, Nanjing 210096, China; 2 Thoracic Surgery Laboratory, The First College of Clinical Medicine, Xuzhou Medical University, Xuzhou 221006, China; 3 School of Medicine, Southeast University, Nanjing 210097, China

**Keywords:** single-cell RNA-seq, spatial transcriptome, Parkinson’s disease, hippocampus

## Abstract

The hippocampus is an important part of the limbic system in the human brain that has essential roles in spatial navigation and cognitive functions. It is still unknown how gene expression changes in single-cell in different spatial locations of the hippocampus of Parkinson’s disease. The purpose of this study was to analyze the gene expression features of single cells in different spatial locations of mouse hippocampus, and to explore the effects of gene expression regulation on learning and memory mechanisms. Here, we obtained 74 single-cell samples from different spatial locations in a mouse hippocampus through microdissection technology, and used single-cell RNA-sequencing and spatial transcriptome sequencing to visualize and quantify the single-cell transcriptome features of tissue sections. The results of differential expression analysis showed that the expression of *Sv2b*, *Neurod6*, *Grp* and *Stk32b* genes in a hippocampus single cell at different locations was significantly different, and the marker genes of CA1, CA3 and DG subregions were identified. The results of gene function enrichment analysis showed that the up-regulated differentially expressed genes *Tubb2a*, *Eno1*, *Atp2b1*, *Plk2*, *Map4*, *Pex5l*, *Fibcd1* and *Pdzd2* were mainly involved in neuron to neuron synapse, vesicle-mediated transport in synapse, calcium signaling pathway and neurodegenerative disease pathways, thus affecting learning and memory function. It revealed the transcriptome profile and heterogeneity of spatially located cells in the hippocampus of PD for the first time, and demonstrated that the impaired learning and memory ability of PD was affected by the synergistic effect of CA1 and CA3 subregions neuron genes. These results are crucial for understanding the pathological mechanism of the Parkinson’s disease and making precise treatment plans.

## 1. Introduction

Parkinson’s disease (PD) is the second most common neurodegenerative disease, which mainly affects people over 60 years old. With the advent of an aging society all over the world, PD increases gradually with the increase of age. At present, people are paying more and more attention to non-motor symptoms in PD, such as cognitive impairment and behavioral disorders. Many patients will eventually suffer from dementia, which has a significant impact on the quality of life [1,2,3]. Previous studies have mainly focused on the substantia nigra and striatum, revealing the pathogenesis of PD through nigrostriatal dopaminergic lesions [4], while less scientific research has been done about the hippocampus. However, with the deepening of people’s understanding of the neuropsychiatric symptoms of PD, it was found that the hippocampus can affect the occurrence of the disease through dopamine and other transmitter systems [5]. Therefore, research on the hippocampus can better explain the mechanism of non-motor symptoms of PD.

In the traditional view, the hippocampus was only considered to be closely related to dementia. However, new data from animal models and human studies have altered this view [6,7,8,9], and the results show that the interaction between dopamine transmission and hippocampal synaptic plasticity plays a key role in memory and behavior [10]. Previous studies have shown that the hippocampus was significantly atrophied in patients suffering from PD, regardless of whether they have dementia [11]. Therefore, we speculate that the hippocampus may have a relationship with the occurrence of PD. At present, new data on cognitive impairment and behavioral disorders of PD also verify the role of the hippocampus in cognitive function and behavior [2,12]. Due to the extremely complex composition of cells in the hippocampus, studying the hippocampus as a unique structure does not facilitate the elucidation of the different mechanisms of cognitive performance [13]. Therefore, this study analyzed the gene expression changes in different positions of the hippocampus using single-cell and spatial transcriptome sequencing technologies, which have scientific and clinical importance for a comprehensive understanding and exploration of the pathogenesis and potential therapeutic targets of PD.

Spatial transcriptome sequencing is a key technology to advance the understanding of brain architectures, reveal the spatial disposition of cells and enable researchers to determine cell interactions and tissue construction to better understand diseases’ mechanisms. For example, LCM-based spatial transcriptome sequencing technology analyzed dopamine neurons in substantia nigra pars compacta and the ventral tegmental area of PD, revealing the differential expression of dopamine neurons in different brain regions [14]. At present, single-cell sequencing has revealed neurons and glial cells types in the brains of PD patients, and determined the functional role of these cell types in the process of dopaminergic neuron degeneration [15]. However, the research on spatial transcription sequencing technology in PD is rarely reported.

Here, we took single-cell and spatial transcriptome sequencing to analyze gene expression changes in single cells at different locations in the hippocampus, enabling the identification and characterization of cellular heterogeneity, which was crucial for understanding the cellular dysregulation associated with PD. Our research results reveal the regulatory mechanism of gene expression changes in single cells at different locations on learning and memory, and identified cell location-specific expression genes, providing potential new markers and therapeutic targets for precise treatment of PD.

## 2. Results

### 2.1. Single Cell Captured

**Figure 1:**
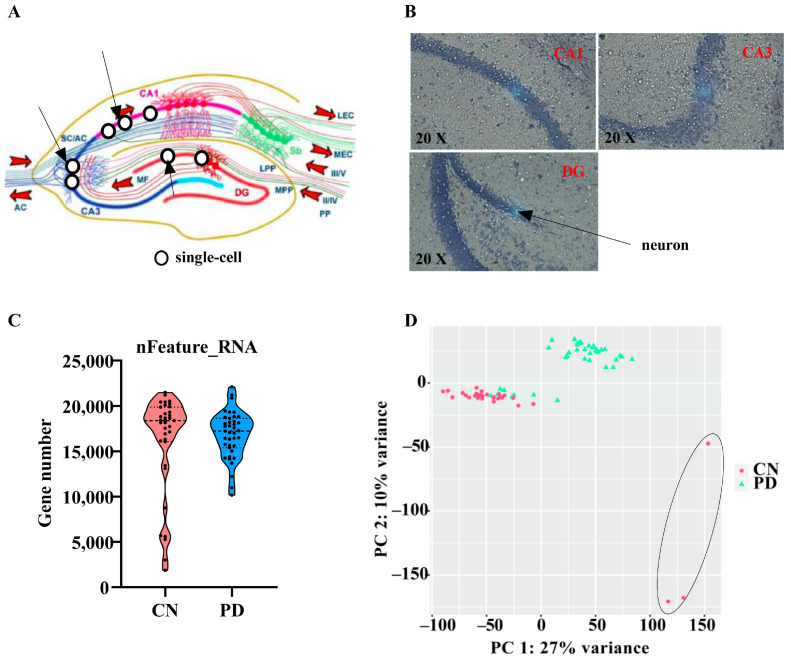
Schematic diagram for obtaining single cells. (**A**) A schematic diagram of the hippocampal formation. (**B**) Brain tissue sections stained with cresol violet. (**C**) Scatterplot illustrating the number of genes in each cell of PD and CN group. (**D**) Principal component analysis of all single-cell samples. CN: control; PD: Parkinson disease.

First, we analyzed the time required for mice to complete the pole test and rotarod test (Figure S1A,B). The results show that the MPTP-induced PD mouse model was successfully induced. In this study, the microdissection technique was used to collect single-cell samples from the hippocampus of the PD and control mouse (Figure 1A), and transcriptome sequencing was performed. To analyze the gene expression changes of single cells in different spatial location of the hippocampus, the neuronal cell bodies were identified by cresyl violet staining (Figure 1B). Seventy-four neuronal cells were obtained from different spatial location of the hippocampus, and a high-quality cDNA library was prepared with the ulRNA-seq library construction method. We obtained an average of 16,600 genes from single cells (Figure 1C). The quality control results showed three single-cell microregion samples deviate significantly from other samples. Therefore, we screened 71 single-cell microregion samples for subsequent analysis (Figure 1D).

### 2.2. Visualization and Bioinformatics Analysis of Tissue Domains

**Figure 2:**
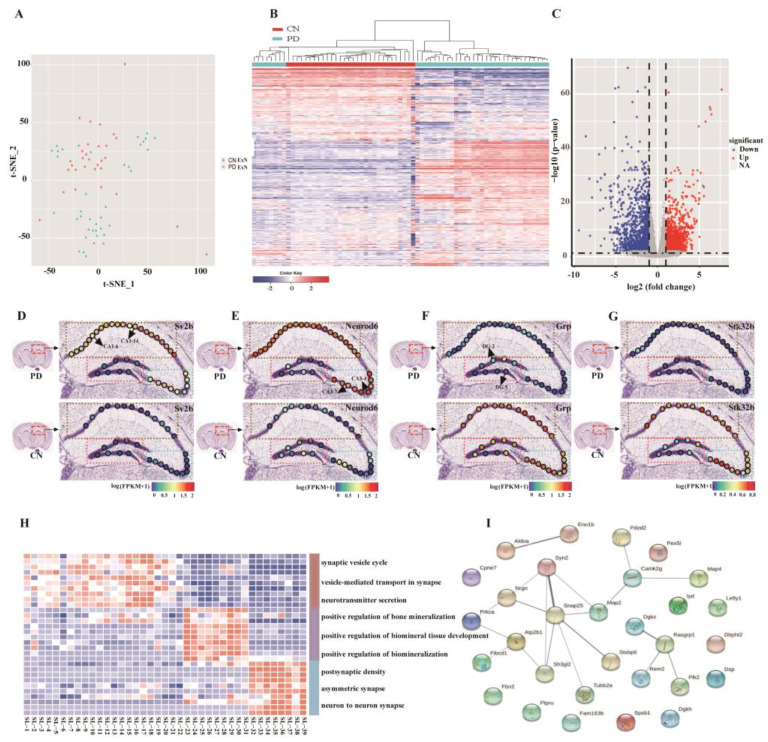
Cell type identification and differential expression analysis. (**A**) Gene expression profiles of single-cell microregion samples in the control group and PD group. (**B**) Single-cell type identification. (**C**) Volcano plot of differential expression of single-cell microregion samples in CN and PD group. (**D**–**G**) showed the expression of *Sv2b*, *Neurod6*, *Grp* and *Stk32b* genes in different spatial locations, respectively. (**H**) Gene expression profiling of the top 10 specific marker genes at different spatial locations. (**I**) Network plot showing marker genes of different spatial locations in the hippocampus. CN: control; PD: Parkinson disease.

To analyze the cell types of single-cell microregion samples, we performed an unsupervised cluster analysis of gene expression profiles in 71 single cells. All single-cell microregion samples were visualized by t-distributed stochastic neighbor embedding (t-SNE). All single-cell microregion samples were annotated according to the expression of cell type specific marker genes, and it was found that all single cells belong to excitatory neurons (Figure 2A). The heatmap showed that the gene expression patterns were different between the PD and control group (Figure 2B), and 2677 differentially expressed genes (DEGs) were screened (Figure 2C). In order to intuitively reflect the transcriptome characteristics at different spatial locations, genes with the largest expression differences were analyzed, namely *Sv2b*, *Neurod6*, *Grp* and *Stk32b*. The results showed that the expression of *Sv2b*, *Neurod6*, *Grp* and *Stk32b* in single cells of different regions of the hippocampus in the control group was different. Compared with the control group, *Sv2b* and *Neurod6* expression were significantly increased in CA1 and CA3 regions of PD mice, while *Grp* and *Stk32b* expression were significantly reduced in single cells of different regions of PD mice. Figure 2D–G provide an example of cluster mapping and identification, revealing the heterogeneity of *Sv2b*, *Neurod6*, *Grp* and *Stk32b* expression at different spatial locations.

In addition, 10 marker genes specifically expressed were screened from 39 different spatial locations in the hippocampus of PD mouse, and GO enrichment analysis was performed. The result shows that single-cell microregion samples at different locations were involved different functional (Figure 2H). A protein interaction network was constructed with the screened marker genes, and a transcription factors regulatory network was constructed to reveal the interaction of genes between different locations (Figure 2I). Among them, the interaction between *Syn2* and *Snap25* is stronger, and the transcription of *Camk2g* is regulated by *Map2* (Figure 2I).

**Figure 3:**
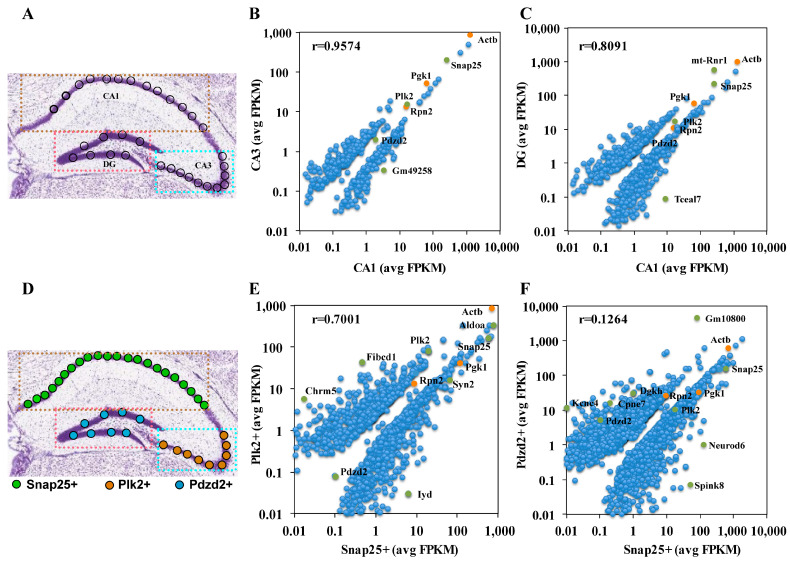
Gene expression profile analysis of tissue domain. (**A**) The single-cell locations of the three subregions in the control group are shown. (**B**) Scatterplot of gene expression correlation between CA1 and CA3 subregions. (**C**) Scatterplot of gene expression correlation between CA1 and DG subregion. (**D**) The spatial expression of marker gene maps of different tissue domains in the hippocampus of Parkinson’s disease. (**E**) Analysis and comparison of differentially expressed genes between Snap25+ expression sites and Plk2+ expression sites. (**F**) Analysis and comparison of differentially expressed genes between Snap25+ expression sites and Pdzd2+ expression sites.

Spatial transcriptome sequencing analysis can be used to define the transcriptome of specific regions. To assess transcriptome characteristics, we analyzed gene expression correlations in single cells at different locations. Analysis between adjacent regions revealed very similar expression profiles, with fewer DEGs (Figure 3A,B). In contrast, comparison to more distant regions revealed different gene expression profiles, and there were more DEGs (Figure 3A,C). The figure shows the differential changes of stable housekeeping genes and specific expressed genes. It is very valuable to explore the gene expression patterns of cell populations or tissue domains that can be identified by specifically expressing genes. Features were selected on the basis of marker genes *Snap25*, *Plk2* and *Pdzd2* in three distinct tissue regions. Figure 3D shows the features distribution of specific genes at different spatial locations in PD. The results showed that the gene expression correlation between *Snap25* and *Plk2* specific expression regions was 0.7001 (Figure 3E), while the gene expression correlation coefficient between *Snap25* and *Pdzd2* specific expression regions was 0.1264 (Figure 3F), which revealed specific transcriptomes defined by these specific marker maps. Therefore, we can determine the spatial region to which a single cell belongs according to the specifically expressed genes.

### 2.3. Analysis of Single-Cell Transcriptome Data in Three Hippocampus Subregions

**Figure 4:**
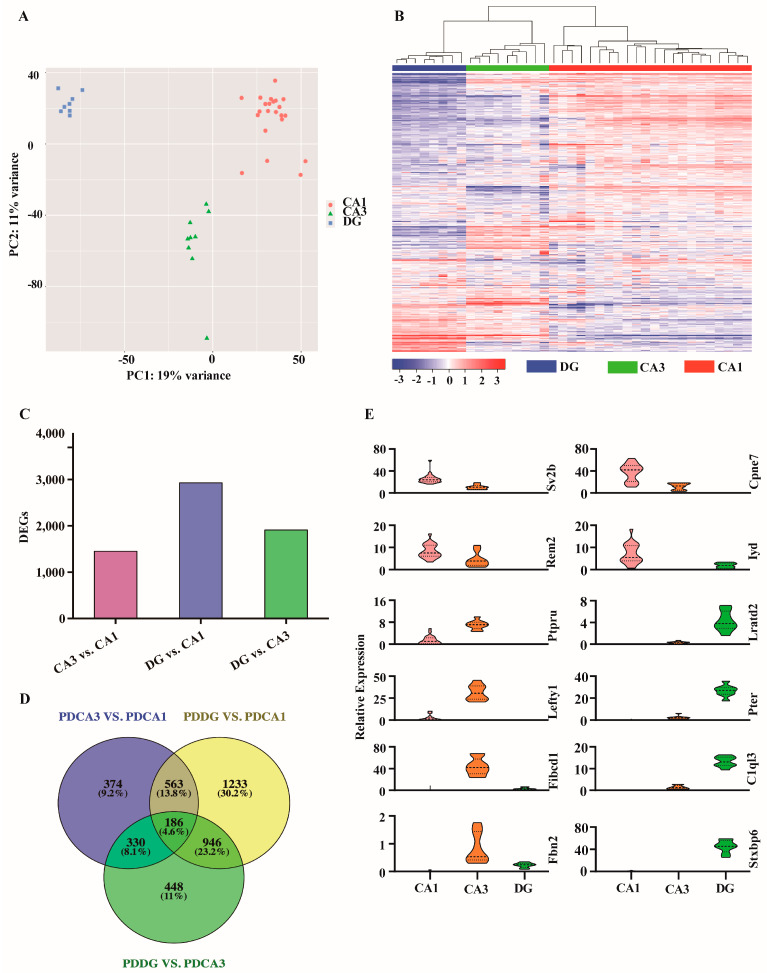
Gene expression characteristics in the hippocampus of PD. (**A**) Principal component analysis of single-cell samples from different subregions. (**B**) Heatmap of expression of all genes in single cells of different subregions. (**C**) Differential expression analysis between different subregions. (**D**) Co-expression analysis of differentially expressed genes between different subregions. (**E**) Analysis of specific highly expressed genes in CA1, CA3 and DG subregions.

We used single-cell resolution spatial transcriptome sequencing to analyze the gene expression patterns between CA1, CA3 and DG subregions in a PD mouse, and identified the differences between hippocampal circuit transcriptional profiles. The result of PCA analysis showed that the three subregions were separated obviously, suggesting the importance of conducting hippocampal subfield-specific analyses (Figure 4A). We used hierarchical clustering to examine gene expression profiles generated. The results showed that there were differences in gene expression patterns in single-cell microregion samples of different subregions (Figure 4B). Compared with CA1 subregion, we identified 1453 and 2928 DEGs in CA3 and DG subregion of PD group, respectively (Figure 4C). Compared with the CA3 subregion, 1910 DEGs were detected in the DG subregion, and there were 186 co-expressed differential genes in the three subregions (Figure 4D). In addition, we also analyzed the trajectories of gene expression changes in the three subregions. The top 12 DEGs were selected from the co-expressed differential genes for analysis of their expression in the three subregions. Among them, *Sv2b*, *Rem2*, *Cpne7* and *Iyd* were highly expressed in the CA1 subregion; *Ptpru*, *Lefty1*, *Fibcd1* and *Fbn2* were highly expressed in the CA3 subregion; and *Lratd2*, *Pter*, *C1ql3* and *Stxbp6* were highly expressed in the DG subregion (Figure 4E). We selected three DEGs from DG subregion for RT-PCR detection. The results showed similar changing trends between the qPCR verification and RNA sequencing results (Figure S2), which indicate the reliability of the sequencing analysis results.

**Figure 5:**
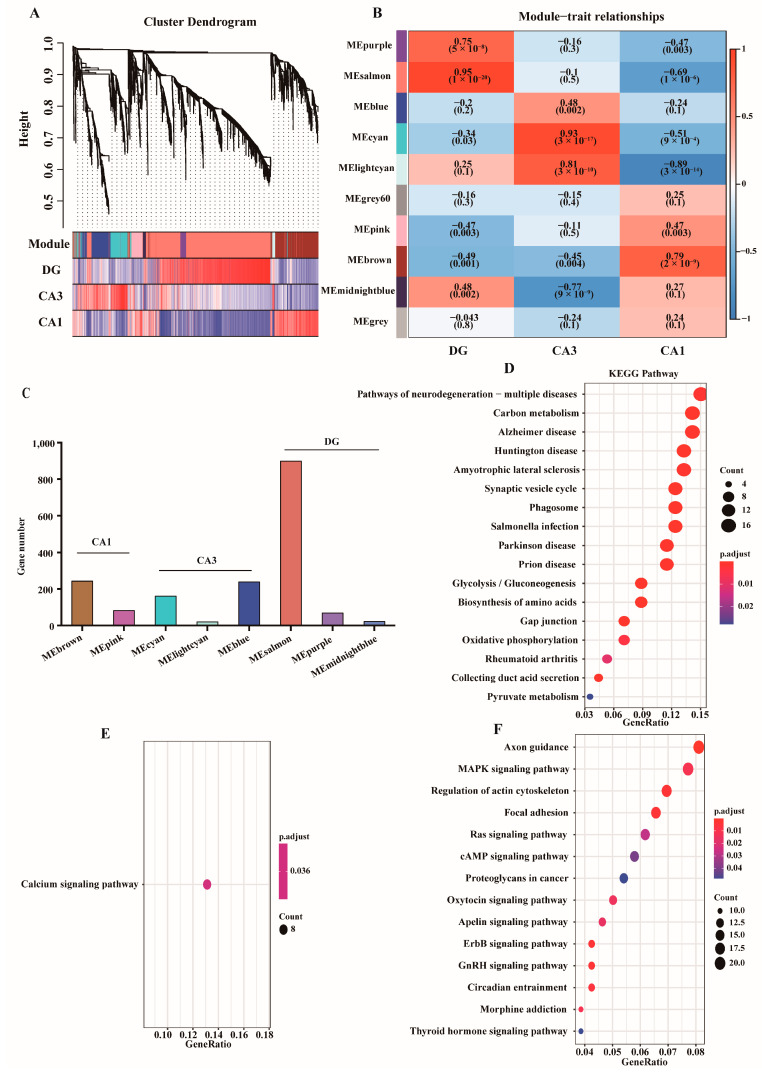
Construction of the co-expression network. (**A**) Clustering dendrogram of all single-cell samples. (**B**) Heatmap of the correlation between the module eigengenes and three subregions of PD. (**C**) The number of genes detected by different modules. (**D**) KEGG pathway analysis of brown module. (**E**) KEGG pathway analysis of cyan module. (**F**) KEGG pathway analysis of salmon module.

Differentially expressed genes can be used to assess the expression levels of genes in different disease states, providing potential therapeutic targets for PD. However, the polygenic nature of PD makes it difficult to draw relevant conclusions from functional lists. We cluster genes into modules through WGCNA analysis, in which the brown module is significantly associated with CA1 subregion (r = 0.95), the cyan module is significantly associated with CA3 subregion (r = 0.93) and the salmon module is significantly associated with DG subregion (r = 0.79) (Figure 5A,B). Figure 5C shows the number of genes contained in each module. Therefore, brown, cyan and salmon modules were selected as clinically important modules for further analysis.

To evaluate the interaction network of all of the associated DEGs in brown, cyan and salmon modules, we performed GO enrichment analysis. In the brown module, significantly enriched GO term included vesicle-mediated transport in synapse, synaptic vesicle cycle, myelin sheath, distal axon, structural constituent of cytoskeleton and GTPase activity (Figure S3A). In the cyan module, significantly enriched GO term included regulation of membrane potential, glutamate receptor signaling pathway, neuron to neuron synapse, postsynaptic membrane, cation channel activity and ion channel activity (Figure S3B). In the salmon module, significantly enriched GO term included regulation of osteoblast differentiation, neuron to neuron synapse, postsynaptic specialization, Rho GTPase binding and small GTPase binding (Figure S3C). In addition, KEGG analysis show that the brown module was mainly involved in pathways of neurodegeneration—multiple diseases. The cyan module is mainly involved in calcium signaling pathway. The salmon module is mainly involved in axonal guidance pathway (Figure 5D–F).

We calculated the module membership (MM) value of each gene to identify hub genes in the module. The correlation analysis between the gene significance (GS) of the different hippocampus subregions and the MM of the gene in each module was conducted to test whether the MM value was closely related to the hippocampus subregions. The results showed that the correlation coefficient between GS and MM in the CA1 subregion was highest in the brown module (correlation coefficient = 0.54, *p* value = 2.3 × 10^−45^) (Figure S4A). The correlation coefficient between GS and MM in the CA3 subregion was highest in the cyan module (correlation coefficient = 0.13, *p* value = 0.026) (Figure S4B). The correlation coefficient between GS and MM in the DG subregion was highest in the salmon module (correlation coefficient = 0.13, *p* value = 0.026) (Figure S4C). We used three criteria to screen the key hub genes in brown, cyan and salmon modules: GS > 0.6, MM > 0.8, weighted (top 100), and kMe (top 30). Twenty hub genes were screened from the three modules respectively (Table S1), among which the hub genes screened in the CA1 subregion were *Aldoa*, *Tubb2a*, *Syn2*, *Sh3gl2* and *Eno1*; the hub genes screened in the CA3 subregions were *Atp2b1*, *Plk2*, *Map4*, *Pex5l* and *Fibcd1*; and the hub genes screened in the DG subregion were *Stxbp6*, *Pdzd2*, *Camk2g*, *Dgkh* and *Fam163b*. Combined with the functional enrichment analysis results of different module genes, we found that the hub genes screened from the CA1 subregion are mainly involved in synaptic transport and neurodegenerative disease pathways, revealing that these genes play a key role in the pathological regulation of PD in the CA1 subregion. The hub genes of CA3 subregion are mainly involved in the glutamate receptor signaling pathway and calcium signaling pathway. However, only *Stxbp6*, *Pdzd2* and *Camk2g* in the DG subregion were involved in neuron to neuron synapse and the GnRH signaling pathway. Based on the above research results, it was found that the single-cell gene expression changes in the DG subregion of PD were small, which may not be the main region of PD.

## 3. Discussion

According to the existing literature, gene expression changes of hippocampal neuronal cells are associated with various neurodegenerative diseases [16,17,18], thus reducing the cognitive ability of patients. Therefore, studying hippocampal tissue has become a crucial and attractive subject for basic medical research and translational neurobiology. However, the hippocampal neuronal cells are highly heterogeneous [19], and the mechanism of cognitive impairment in PD remain incompletely understood. Therefore, in-depth analysis of the gene expression patterns of cells in different spatial locations in the hippocampus is crucial for understanding the composition and function of cell types in the hippocampus. In this study, the gene expression changes of neuronal cells were analyzed at the single-cell level and spatial location, thereby revealing that single cells at different spatial locations in the hippocampus are involved in important biological functions and pathways in PD, providing evidence for explaining the mechanism of cognitive dysfunction in PD.

To our knowledge, this is the first study to use single-cell and spatial transcriptome sequencing technology to provide important functional insights about the molecular definition of cognitive dysfunction in PD. We first analyzed the cell types of the single cells obtained in this study, and the result showed that all single cells belonged to excitatory neurons. Compared with the control group, a total of 2677 DEGs were detected in the hippocampus single-cell samples of PD, of which *Sv2b*, *Neurod6*, *Snap25* and *Stk32b* were differentially expressed. Previous studies have shown that *Sv2b*, *Neurod6*, *Snap25* and *Stk32b* were differentially expressed in the brains of PD patients, which revealed that they were related to the occurrence of PD [20,21,22,23], but the level of expression of these genes in tissue spatial location is unknown. Although single-cell transcriptome analysis has also shown that *Neurod6* was used as a specific marker for midbrain dopaminergic subpopulations, the spatial location of cell subpopulations has not been studied [23]. In this study, the gene expression pattern of spatial tissue structure was studied by single-cell resolution spatial transcriptome sequencing, and the results showed that the expression of *Snap25*, *Sv2b* and *Neurod6* was significantly increased in single cells of the CA1 and CA3 subregions of the hippocampus. It is well known that CA1 plays a crucial role in memory, especially when re-experiencing memory [24]. According to the existing research results, Neurod6 is related to brain development and cognitive function, and can regulate neural differentiation and neuronal survival [25,26], thereby impairing the memory function innervated by the hippocampus. SNAP25 is a synaptosome-associated protein involved in the regulation of neurotransmitter release [27,28]. Yun et al. reported that LRRK2 may regulate neurotransmitter release via control of *Snap25* by inhibitory phosphorylation and controlling the function of *Snap25* [29]. Overexpression of *Snap25* can cause synaptic dysfunction, thereby reducing learning and memory ability [29]. Consequently, these observations provide important insights on the pathophysiology of cognitive dysfunction in PD.

In addition, WGCNA was used to further analyze gene function in the single-cell samples of different subregions of PD. The results showed that the single-cell samples of the CA1 subregion were mainly involved in vesicle-mediated transport in synapse and neurodegenerative disease-related functions, and *Syn2*, *Sh3gl2*, *Aldoa*, *Tubb2a* and *Eno1* were screened as the key target genes. Among them, *Syn2* and *Sh3gl2* are mainly involved in synaptic transmission and neurotransmitter secretion. It is well known that α-syn aggregation leads to the formation of Lewy bodies [30], which is the main pathological feature of PD. However, Lewy body lesions are unevenly distributed in the hippocampus of patients with PD, mainly in the CA1 and CA2 subregions, while DG and CA3 subregions were free of Lewy-body pathology [31]. Previous studies have shown that excess α-Syn is expressed in oligodendrocyte via vesicle-mediated transport [32], which leads to differential expression of CA1 subregion. This is the first experimental evidence of synaptic dysfunction in the hippocampus, and also reveals that there is a link between cognitive defects in PD and Lewy body pathology in the CA1 subregion of the hippocampus [33,34,35,36]. Aldoa and Eno1 are enzymes related to glycolysis and energy metabolism [37,38], and play an important role in the post-translational modification of PD-related proteins. It suggested that the function of cell CA1 subregion of hippocampus in PD was changed in glycolysis and energy metabolism pathways [39]. These metabolic changes will lead to chronic neuronal dysfunction, mainly manifested as synaptic function changes. Differing from the CA1 subregion, the single-cell samples of the CA3 subregion are mainly involved in the glutamate receptor signaling pathway and calcium ion transport channel, and screened out *Atp2b1*, *Plk2*, *Map4*, *Pex5l* and *Fibcd1* as the key genes of this subregion. Previous studies have shown that *Atp2b1* is a key gene for calcium ion transport (calcium ion dysregulation is involved in the pathogenesis of PD [40]) and plays a fundamental role in synaptic plasticity, synaptic transport, memory and learning [41,42]. *Plk2* is considered to be the main enzyme of α-synuclein phosphorylation and plays a key role in the pathogenesis of PD [43]. While *Map4* is mainly expressed in neural cells [44], its function in the mechanism of PD is still unclear. Therefore, single-cell and spatial transcriptome technology can accurately analyze the location of differentially expressed genes in tissue space, which reveals the importance of tissue-spatial gene expression analysis for PD research. These genes can be used as markers of CA3 subregion of PD. However, *Fibcd1* is highly expressed in the CA3 subregion, which had not been reported in previous studies, so the role of this gene in the disease needs to be further investigated.

Based on the results of gene function enrichment analysis, we found that the decline of learning and memory in PD patients was influenced by the synergy of genes in neurons of the CA1 and CA3 subregions. CA1 subregion neuron cells release neurotransmitters through presynaptic vesicles, which convert electrical signals into chemical signals, thereby activating Ca^2+^ transport channels in single cells in the CA3 subregion. The increase of intracellular free calcium levels gives rise to a range of calcium-dependent vital cellular processes such as impaired plasticity, neuronal degeneration and cognitive impairment [45,46]. However, we found that the gene expression changes in the DG subregion of PD were small, mainly involved in neuron to neuron synapse and synaptic guidance pathways, and screened five key targets gene of *Stxbp6*, *Pdzd2*, *Camk2g*, *Dgkh* and *Fam163b*. In previous studies, attention has not been paid to these genes in the pathological mechanism of PD. In this study, single-cell and spatial transcriptome sequencing technology revealed the importance of these genes in the DG subregion of PD. Due to the limited number of single-cell samples, we should increase the single-cell resolution and the number of samples in spatial locations in future experiments to further improve and verify the accuracy of the data.

## 4. Materials and Methods

### 4.1. Animals

Eight male mice were housed in an SPF-grade room under a 12 h light/dark cycle, fed standard rodent chow and water and randomly divided into a control group and a model group, with four mice in each group. Model group C57Bl/6J mice (8 weeks old) were treated intraperitoneally with MPTP (10 mg/kg, Sigma, St. Louis, MO, USA) for 10 days to induce an acute PD model. The control group mice were injected with the same amount of normal saline. After 10 days, we first conducted the pole test and the rotarod test. After the MPTP-induced PD mouse model was successfully induced, we selected one mouse from PD group and one mouse from control group for spatial transcriptome study. All experimental procedures were reviewed and approved by the Ethics Committee of Zhongda Hospital Southeast University.

### 4.2. Single Cell Collection

Firstly, mouse brain tissue section was taken on a microtome at 20 µm thickness, then tissue section was mounted on glass slides with polyethylene naphthalate membranes. Prior to microdissection, it is necessary to cresyl violet stain the cryosection for better visualization to determine the DG, CA3 and CA1 orientation of hippocampal subfields for dissection. Then, single cells were dissected with a laser capture microdissection (LCM) system using the ultraviolet (UV) laser to cut out single-cell sample in the DG, CA3 and CA1 regions. We obtained 39 single cells from the PD group and 35 single cells from the control group.

### 4.3. Single-Cell Library Preparation

Single-cell library preparation was performed using ulRNA-seq protocol [47]. First, RNA templates were denatured at 72 °C for 3 min, and immediately placed on ice afterward. Second, template switching first-strand cDNA synthesis was performed using a 5′-biotinylated TSO oligo. Third, PCR pre-amplification was performed directly after reverse transcription, and then the cDNA product was purified with 0.8 × Ampure XP beads. Fourth, cDNA concentration was measured using the Qubit dsDNA Assay Kit (Thermo Fisher Scientific, Waltham, MA, USA). Fifth, 1 ng of cDNA was used for the tagmentation reaction carried out with One-step DNA Lib Prep Kit for Illumina (ABclonal, Wuhan, China). Finally, cDNA library fragment distribution was detected with the Agilent 4200 High Sensitivity DNA Assay Kit (Agilent Technologies, Palo Alto, CA, USA). According to the detection results, the quality of the cDNA library was determined, and the subsequent cDNA library was sequenced on the Illumina HiSeq X10 PE150 platform (Illumina Inc., San Diego, CA, USA).

### 4.4. Bioinformatics Analysis

Firstly, the quality control analysis of the raw data is carried out, and the reads containing adapter or poly-N sequences and low-quality reads are removed to generate clean data for quantitative analysis of gene expression. Then, clean data were aligned to the mouse reference sequences by Hisat2 (v2.0.5) software. Finally, featureCounts v1.5.0-p3 software was used to count the reads numbers mapped to each gene, and then the fragments per kilobase of transcript per million (FPKM) of each gene was calculated according to the length of the gene. The similarity between samples was evaluated by principal components analysis (PCA), cluster analysis and correlation analysis. The DEGs between different groups were analyzed with the DESeq2 R package (1.16.1). Gene ontology (GO) enrichment analysis and Kyoto encyclopedia of genes and genomes databases (KEGG) enrichment analyses were performed using the clusterProfile R package. The WGCNA R package was used to analyze the weighted gene co-expression network analysis (WGCNA) of three hippocampal subregions.

## 5. Conclusions

In summary, we revealed the spatially mapped transcriptomic signature of the hippocampus in PD mice by single-cell and spatial transcriptome sequencing techniques, discovered that transcriptional regulation between cells in different locations affects cognitive dysfunction and screened some key marker genes. Among them, the up-regulated differentially expressed genes *Tubb2a*, *Eno1*, *Atp2b1*, *Plk2*, *Map4*, *Pex5l*, *Fibcd1* and *Pdzd2* were mainly involved in neuron to neuron synapse, vesicle-mediated transport in synapse, the calcium signaling pathway and neurodegenerative disease pathways, while the down-regulated differentially expressed genes *Sh3gl2*, *Aldoa*, *Stxbp6* and *Camk2g* are mainly involved in ATP metabolism and the GnRH signaling pathway. The key genes identified in this study are expected to become new targets for disease treatment, providing new ideas for in-depth exploration of the pathogenesis of PD.

## Data Availability

The scRNA-seq datasets are available at NCBI project PRJNA899134 (https://www.ncbi.nlm.nih.gov/, accessed on 7 November 2022).

## Supplementary Materials

The following supporting information can be downloaded at: https://www.mdpi.com/article/10.3390/ijms24031810/s1.

## Abbreviations

PD	Parkinson’s disease
OCT	optimum cutting temperature
LCM	laser capture microdissection
FPKM	fragments per kilobase of transcript per million
PCA	principal components analysis
DEGs	differentially expressed genes
GO	Gene ontology
KEGG	Kyoto encyclopedia of genes and genomes databases
WGCNA	weighted gene co-expression network analysis
t-SNE	t-distributed stochastic neighbor embedding
MM	module membership
GS	gene significance
