# Supplementary material for: Spatial Transcriptome Profiling of Mouse Hippocampal Single Cell Microzone in Parkinson’s Disease
Source: Int J Mol Sci. 2023 Jan 17;24(3):1810. doi: 10.3390/ijms24031810 (PMC9915078; doi:10.3390/ijms24031810)
Supplement: Supplementary file 1 [file ijms-24-01810-s001.zip › Supplementary Figure.pdf]

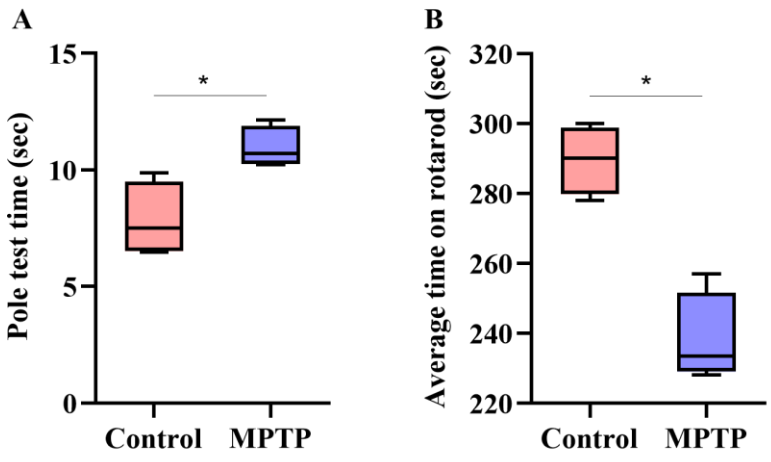

Fig. S1. Measuring the exercise capacity of 1-methyl-1,2,3,6-tetrahydropyridine (MPTP)-induced model mice in a (A) pole test and (B) rotarod test. * indicates a *p*-value < 0.05.

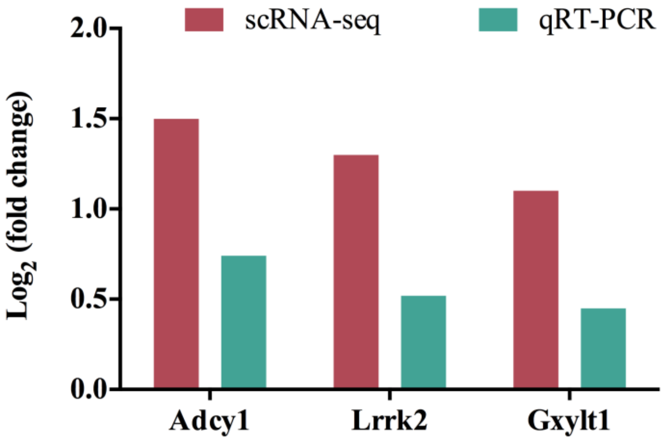

Fig. S2. Quantitative real-time PCR validation of the selected DEGs.

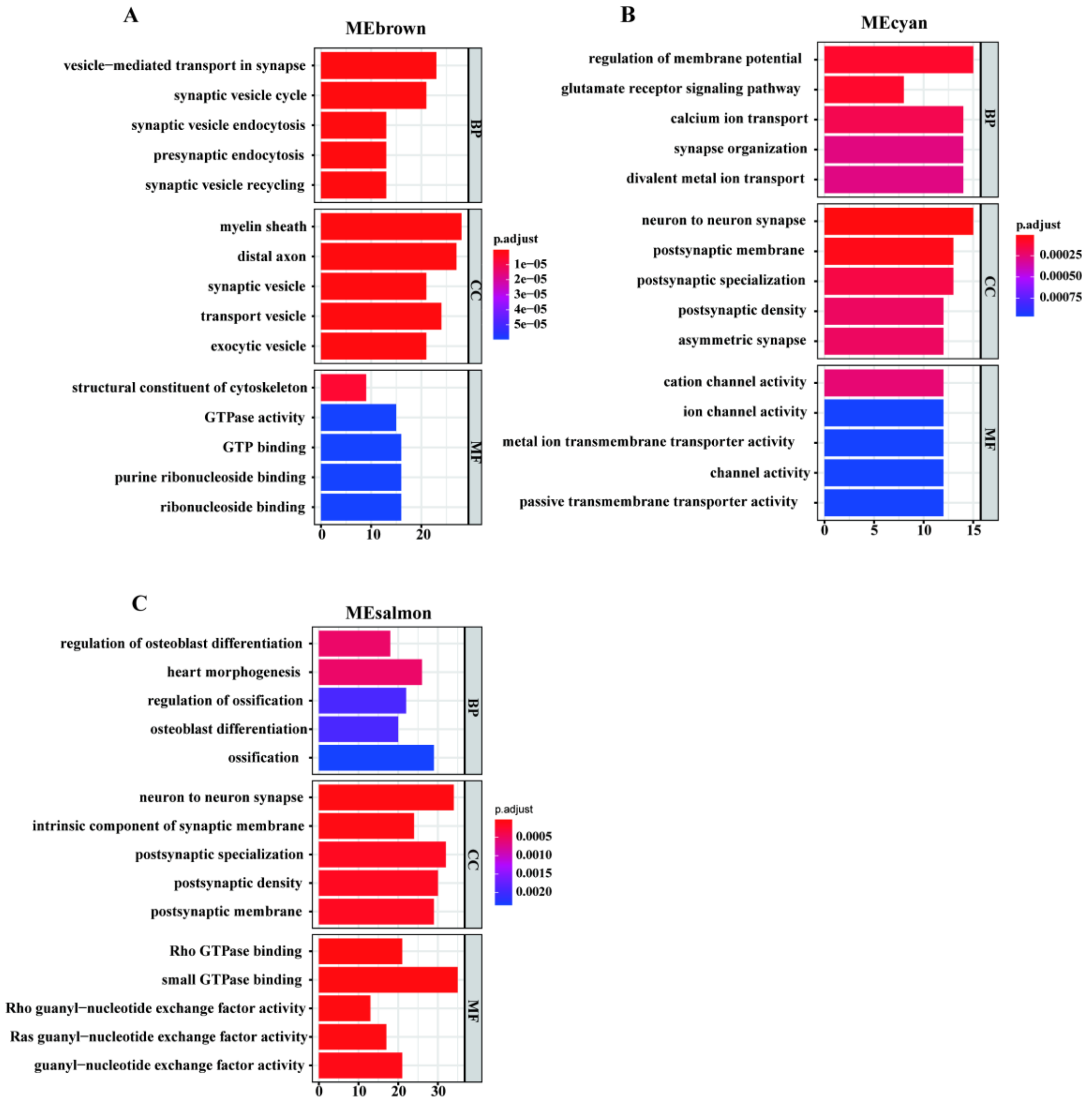

Fig S3. GO enrichment analysis of differentially expressed genes. (A) The GO enrichment analysis of brown module. (B) The GO enrichment analysis of cyan module. (C) The GO enrichment analysis of salmon module.

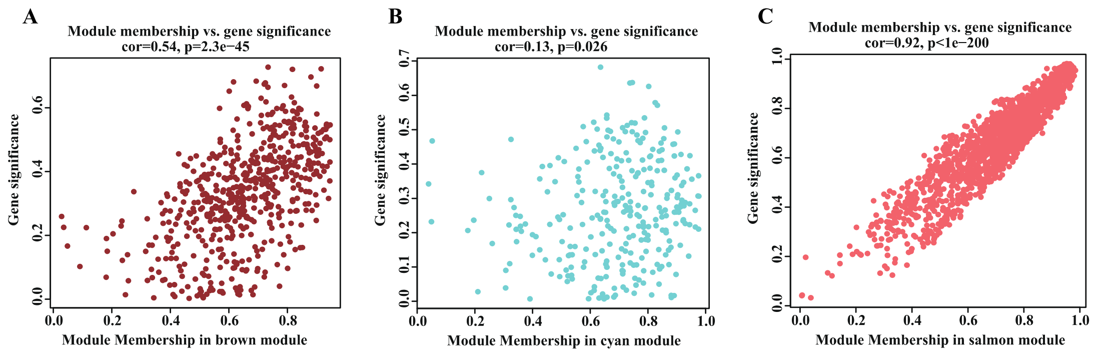

Fig S4. Correlation analysis between GS and MM. (A) Scatter plot of GS and MM in brown module. (B) Scatter plot of GS and MM in cyan module. (C) Scatter plot of GS and MM in salmon module.
