# Supplementary material for: Spatial Transcriptome Profiling of Mouse Hippocampal Single Cell Microzone in Parkinson’s Disease
Source: Int J Mol Sci. 2023 Jan 17;24(3):1810. doi: 10.3390/ijms24031810 (PMC9915078; doi:10.3390/ijms24031810)
Supplement: Supplementary file 1 [file ijms-24-01810-s001.zip › Supplementary Table.pdf]

Table S1 Hub genes screened in brown, cyan and salmon modules.

MEbrown		MEcyan		MEsalmon	
Gene	kMe 值	Gene	kMe 值	Gene	kMe 值
Rab6b	0.94	*Map4*	0.974	*Cnih3*	0.982
Eno1	0.94	*Plk2*	0.973	*Marcks*	0.977
Cadps	0.94	*Fibcd1*	0.973	*Dgkh*	0.975
Aldoa	0.94	*Gpr161*	0.971	*Fam163b*	0.975
Slc6a17	0.938	*Pex5l*	0.962	*Pkig*	0.971
Cabp1	0.935	*Mpped1*	0.962	*Ralgapa2*	0.969
Tubb2a	0.933	*Ccdc88c*	0.950	*C1ql3*	0.968
Synj1	0.932	*Pid1*	0.943	*Syne1*	0.964
Reep5	0.931	*Atp2b1*	0.942	*Nedd4l*	0.964
Stmn2	0.931	*Kcnh7*	0.942	*Pitpnm2*	0.963
Pgam1	0.929	*Gm37443*	0.939	*Arhgap20*	0.962
Tuba4a	0.925	*Man1a*	0.933	*Plk5*	0.962
Ogfrl1	0.923	*Tenm3*	0.927	*Trpc6*	0.962
Atp6v0c	0.922	*Wfs1*	0.927	*Sorbs2*	0.962
Selenow	0.921	*Galntl6*	0.922	*Adcyap1r1*	0.961
Syn2	0.918	*Ntm*	0.921	*Tnik*	0.961
Ap2s1	0.918	*B230110G15Rik*	0.921	*Pde1b*	0.961
Sh3gl2	0.916	*Ndst3*	0.920	*Stxbp6*	0.960
Stmn3	0.913	*Sorcs3*	0.915	*Igfbp5*	0.960
Cpe	0.913	*Zdhhc2*	0.915	*Nfia*	0.959
Chgb	0.909	*Htr1a*	0.913	*Sv2c*	0.959
Tuba1b	0.909	*Ptpru*	0.912	*Btbd9*	0.959
